# Supplementary material for: Intestinal Atp8b1 dysfunction causes hepatic choline deficiency and steatohepatitis
Source: Nat Commun. 2023 Nov 21;14:6763. doi: 10.1038/s41467-023-42424-x (PMC10663612; doi:10.1038/s41467-023-42424-x)
Supplement: Supplementary file 1 — Supplementary Information [file 41467_2023_42424_MOESM1_ESM.pdf]

Intestinal Atp8b1 dysfunction causes hepatic choline deficiency and steatohepatitis

Ryutaro Tamura[1,12], Yusuke Sabu[1,12], Tadahaya Mizuno[1], Seiya Mizuno[2], Satoshi Nakano[3], Mitsuyoshi Suzuki[3], Daiki Abukawa[4], Shunsaku Kaji[5], Yoshihiro Azuma[6], Ayano Inui[7], Tatsuya Okamoto[8], Seiichi Shimizu[9], Akinari Fukuda[9], Seisuke Sakamoto[9], Mureo Kasahara[9], Satoru Takahashi[2], Hiroyuki Kusuhara[1], Yoh Zen[10], Tomohiro Ando[11], Hisamitsu Hayashi[1]*

[1]Laboratory of Molecular Pharmacokinetics, Graduate School of Pharmaceutical Science, The University of Tokyo, Tokyo, Japan

[2]Laboratory Animal Resource Center and Trans-Border Medical Research Center, University of Tsukuba, Ibaraki, Japan

[3]Department of Pediatrics, Juntendo University Graduate School of Medicine, Tokyo, Japan

[4]Department of Gastroenterology and Hepatology, Miyagi Children's Hospital, Miyagi, Japan

[5]Department of Pediatrics, Tsuyama-Chuo Hospital, Okayama, Japan

[6]Department of Pediatrics, Yamaguchi University Graduate School of Medicine, Yamaguchi, Japan

[7]Department of Pediatric Hepatology and Gastroenterology, Saiseikai Yokohama City Eastern Hospital, Kanagawa, Japan

[8]Department of Pediatric Surgery, Kyoto University Hospital, Kyoto, Japan

[9]Organ Transplantation Center, National Center for Child Health and Development, Tokyo, Japan

[10]Institute of Liver Studies, King's College Hospital & King's College London, London, UK

[11]Axcelead Drug Discovery Partners, Inc, Fujisawa, Kanagawa, Japan

[12] These authors contributed equally: Ryutaro Tamura, Yusuke Sabu

E-mail: hayapi@mol.f.u-tokyo.ac.jp

Supplementary Methods

Preparation of brush border membrane fractions of IEC

The brush border membrane fractions of IEC were prepared by OptiPrep^{TM} (Serumwerk Bernburg AG, Bernburg Germany) gradients according to manufacturer's instruction. Briefly, IEC were harvested from the SI with a glass slide and homogenized in Tris buffer (20 mM, pH 7.5) with 2 mM DTT, 1 mM EGTA, and 1.006 mM $CaCl_2$. The homogenates were centrifuged at 3,000×g for 5 min to remove unbroken cells, nuclei, and debris. The supernatant was loaded on a 3-30 % iodixanol gradients from OptiPrep^{TM} with Hepes buffer (20 mM, pH 7.2) containing 150 mM NaCl and 20 mM NaF. The gradients were prepared using Gradient Station (BioComp Instruments, Fredericton, Canada) according to manufacturer's instruction. After centrifugation at 90,000×g for 90 min at 4 °C in an SW-41 Ti rotor (Beckman Coulter, Brea, CA), twenty fractions were collected by Gradient Station. The prepared specimens were analyzed by immunoblotting with Atp1a1 (Santa Cruz Biotechnology, sc-21712, clone: C464.6, 1:200) and villi (Cell Signaling Technology, 2369, 1:2000), a marker of basolateral and brush border membranes, respectively, to identify the fractions corresponding to the brush border membranes. The brush border membranes (fractions 13–15) were subjected to target lipidomic analysis.

Pharmacokinetic study on oral administration of [methyl-^{3}H]-choline chloride

Male 8-week-old Atp8b1$^{Tax\text{-}iIEC\text{-}KO}$ mice (line #12) and littermate Atp8b1$^{flox/flox}$ mice were treated daily with 1mg of TAX intraperitoneally for 5 days and then used for the experiment on day 6. The mice were fasted for 12 h and orally administered a choline chloride solution (50 mg/kg body weight; FUJIFILM Wako Pure Chemical) containing [methyl-^{3}H]-

choline chloride (150 μCi/kg body weight; PerkinElmer, Waltham, MA). Blood samples were collected from the tail vein into EDTA-coated tubes at 30 and 60 min after the dosing. The plasma was separated by centrifugation at 1700×g for 15 min and stored at −80 °C. The mice were sacrificed 120 min after the dosing, and their liver and SI were excised. The SI lumen was flushed with cold PBS containing 0.5 mM taurocholic acid, and the flushing flow was collected. Each sample was processed using SOLVABLE (PerkinElmer) according to the manufacturer's protocol. The amount of radioactivity was measured by a liquid scintillation counter (Tri-Carb 3110TR, PerkinElmer), and the data were expressed as disintegrations per minute (DPM).

Metabolomic and lipidomic analysis

HILIC/MS/MS analysis

Metabolites were extracted from plasma, liver, and IEC with methanol. After centrifugation at 20,000×g for 5 min at 4 °C, the supernatant was mixed with 400 mM ammonium formate at a ratio of 19:1. The mixture was centrifuged at 20,000×g for 5 min at 4 °C. The supernatant was applied to an LC/MS/MS system, which consisted of a UHPLC Nexera liquid chromatography system (Shimadzu Co., Kyoto, Japan) and a 5500QTRAP mass spectrometer (AB Sciex Pty. Ltd., Toronto, Canada). The analytes were separated through a ZIC-cHILIC column (2.1×100 mm, 3 μm; Merck Millipore) at a temperature of 30 °C with a gradient elution of the mobile phases, 10 mM ammonium formate aqueous solution and acetonitrile at a flow rate at 0.4 mL/min. The eluent was ionized using electrospray ionization and scanned with multiple reaction monitoring (MRM) mode, as described previously [1]. The MRM data were processed using MultiQuant 3.0 (AB

Sciex). Each MRM peak was assigned to a target molecule in comparison with its authentic standard. For identified metabolites, MRM peak areas were calculated and relative comparisons were made between samples.

GC/MS/MS analysis

Metabolites were extracted from plasma, liver, and IEC with methanol. After centrifugation at 20,000×g for 5 min at 4 °C, internal standards labelled with stable isotopes were added to the supernatant and dried under a stream of nitrogen gas. The dried samples were derivatized by oximation and trimethylsilylation. The derivatized metabolites were injected into an Agilent 7890A series gas chromatography system. Chromatographic separation was performed in a J&W Scientific DB-5MS-DG column (30 m×0.25 mm i.d., df = 0.25 µm; Agilent Technologies Inc., Santa Clara, CA) by a temperature gradient with helium gas flow at a rate of 1 mL/min. Eluted metabolites were introduced into an Agilent 7010B triple-quadrupole mass spectrometer for electron impact ionization and scanned in MRM mode. The MRM data were processed by MassHunter (Agilent Technologies Inc.). For identified metabolites, MRM peak areas were calculated and relative comparisons were made between samples.

Non-targeted lipidomic analysis

Lipidomic analysis was performed as previously described [2]. A sample was extracted with ethanol supplemented with 0.002% butylated hydroxytoluene and centrifuged at 21,500×g for 5 min. The supernatant was injected into a CAPCELL PAK ADME column (2.1×100 mm, 2.7 µm; Shiseido, Kyoto, Japan) and maintained at a temperature of 60 °C,

and the lipids were separated by gradient elution of aqueous mobile phase (MilliQ water with 0.01% acetic acid, 1 mM NH_3, and 10 μM EDTA-2Na) and organic mobile phase [0.001% acetic acid and 0.2 mM NH_3 in ethanol/isopropanol (1:1)], with the flow rate set to 0.7 mL/min. The eluents were introduced to a Q Exactive HF-X Mass Spectrometer (Thermo Fisher Scientific). Their mass spectra were acquired in the data-dependent mode. Precursor and product ion spectra by higher-energy collisional dissociation were scanned with the orbitrap analyzer at a resolution of 120,000 and 7,500 full width at half maximum at 200 m/z. The raw LC/MS data were processed by Expressionist Refiner MS software (ver. 8.2; Genedata AG, Basel, Switzerland). Each MS peak was compared with the in-house lipid database including information on retention time, exact mass, and the preferred adduct ion species, and its structure was estimated. For identified lipids, MS peak areas were calculated and relative comparisons were made between samples.

Targeted lipidomic analysis of lysophospholipids

Analysis was performed with the targeted lipidomic method according to the previous report [3]. Lipids were extracted from a sample with methanol in the presence of internal standards. The extract was further purified with chloroform/water (5:4, v/v). The organic layer was collected into a clean tube and dried under nitrogen stream. After its reconstitution with methanol, the sample was injected into a supercritical fluid chromatography/triple-quadrupole mass spectrometry system consisting of an ACQUITY Ultra-Performance Convergence Chromatography system and a Xevo TQ-XS triple-quadrupole mass spectrometer coupled with an electrospray ionization probe (Waters, Milford, MA). Lipids were loaded on an ACQUITY UPC2TM Torus diethylamine column (100 × 3.0 mm inner diameter (i.d.), particle size: sub-1.7

μm, Waters) using an autosampler and eluted under the gradient of CO_2 and ammonium acetate/methanol/water (0.001:95:5, w/v/v, modifier). The eluates were ionized with modifier and scanned with selected reaction monitoring mode. MS data obtained was processed with TargetLynx (Waters). The concentration of each lysophospholipid in a sample was calculated with a single-point calibration.

Visualization of lipid-subtype enrichment in lipidomic analysis

The result of lipidomic analysis was evaluated using Kolmogorov–Smirnov (KS) running sum statistic to determine the enrichment of lipid subtypes. For each tissue, the change in each lipid content between Atp8b1$^{\text{IEC-KO}}$ mice and Atp8b1$^{\text{flox/flox}}$ mice was scored as follows:

$$score_{l,i} = \frac{x_{l,i} - \mu_{control,l}}{\sigma_{control,l}}$$

where $x_{l,i}$ is the amount of lipid l in the ith sample of the Atp8b1$^{\text{IEC-KO}}$ mouse group, and $\mu_{control,l}$ and $\sigma_{control,l}$ are mean and standard deviation of the amount of lipid l in the Atp8b1$^{\text{flox/flox}}$ mouse group. KS statistics were calculated for each lipid subtype and are plotted as running sum at the top of Figure 4A and E. The middle heatmap and the bottom barcode represent the magnitude of difference in each lipid content and the positions of the lipids classified into the indicated subtypes, respectively.

Pathway-level analysis for choline metabolites

Pathway-level analysis for choline metabolites was performed using the generally applicable gene set enrichment

(GAGE) method [58]. Briefly, metabolites in the choline pathway and overall metabolites were subjected to Welch's t-test, comparing all sample combinations of Atp8b1$^{IEC\text{-}KO}$ mice (line #12) and the littermate Atp8b1$^{flox/flox}$ mice. Negative log-sum values of resultant p-values were adjusted based on control group dependencies, and the integrated p-value was computed on a Gamma distribution with K degrees of freedom and a scale of 1.0, where K represents the number of samples of Atp8b1$^{IEC\text{-}KO}$ mice (line #12). Data was analyzed with scipy (ver 1.11) modules of python 3.

Measurement of choline metabolites

The plasma concentrations of choline, betaine, and DMG were determined with an LC/MS/MS assay. The sample was mixed with 98-fold volume of ethanol and 1 volume of internal standard (IS) solutions [choline (Toronto Research Chemicals, Toronto, Canada), phosphocholine (Toronto Research Chemicals) at 10 µmol/L, and betaine (Sigma-Aldrich) at 1 µmol/L in water]. After vortex mixing, samples were centrifuged at 21,500×g for 5 min at 4 °C. The supernatant was injected into a UHPLC Nexera liquid chromatography system (Shimadzu Co.) equipped with an Ascentis Express HILIC column (2.1×50 mm, 2.7 µm, Merck KGaA, Darmstadt, Germany) and maintained at 40 °C; analytes and IS were separated by gradient elution of mobile phase A, 10 mmol/L CH_3COONH_4 in water, and mobile phase B, acetonitrile/water mixture (99:1, v/v). The gradient was as follows: 0–2 min, 82.5% B; 2–4 min, 82.5% to 5% B; and 4–7 min, 5% B, with a flow rate of 0.6 mL/min. The eluate was directly introduced under electrospray ionization in positive ion mode on a 5500QTRAP mass spectrometer (AB Sciex). All target molecules were scanned in selected reaction monitoring (SRM) mode; the conditions are listed in Supplementary Table 7.

For the calibration curve, working solutions were prepared by dilution of a stock solution to the concentrations of 0.5, 1, 2.5, 5, 10, 25, 50, 100 and 250 μmol/L for choline and betaine, and 0.05, 0.1, 0.25, 0.5, 1, 2.5, 5, 10 and 25 μmol/L for DMG in ethanol. Ten microliters of a working solution was mixed with 10 μL of the IS solution and water, and 970 μL of ethanol and then processed as described above. The linear calibration curve was drawn with a weighting of 1/× and confirmed by assessing the accuracy within a range of ± 20%. The SRM data were processed by MultiQuant 3.0.2 (AB Sciex).

Measurement of methionine metabolites

The plasma concentrations of methionine, homocysteine, SAM, and S-adenosyl homocysteine were determined with an LC/MS/MS assay. The samples were homogenized with methanol and derivatized in the presence of β-(4-hydroxyphenyl)ethyl iodoacetamide (Toronto Research Chemicals) and internal standards [homocysteine (3,3,4,4,-D4) (Cambridge isotope laboratories, Andover, MA) at 10 μmol/L in water]. Reactants were centrifuged at 21,500×g for 5 min at 4 °C. The supernatant was injected into the same LC/MS/MS system as for the metabolomic analysis. The analytes and IS were separated through a Luna column (C18(2), 2.1×100 mm, 3 μm, 100Å, Phenomenex) at a temperature of 40 °C with a gradient elution of the mobile phase A, 0.5 mol/L heptafluorobutyric acid (Tokyo Chemical Industry, Tokyo, Japan)/acetic acid/distilled water (1:0.1:100, v/v/v), and mobile phase B, methanol/acetonitrile/acetic acid (50:50:0.1, v/v/v). The gradient was as follows: 0–6 min, 1% B; 6–8.5 min, 95% B; and 8.5–11 min, 1% B, with a flow rate of 0.4 mL/min. The eluent was directly introduced under electrospray ionization in positive ion mode and scanned with MRM mode; the conditions are

listed in Supplementary Table 8. The MRM data were processed by MultiQuant 3.0.2. A plasma concentration of each metabolite was quantitated with a calibration curve prepared with an authentic standard.

Lpcat activity assay

Acyltransferase activity was determined by measuring the incorporation of 18:1 (n9) oleoyl coenzyme A (Avanti Polar Lipids, Alabaster, AL) into 12:0 LysoNBD-PC (Avanti Polar Lipids) as described previously [4]. Briefly, 1×10^6 IEC were prepared from 4-week-old Atp8b1$^{IEC\text{-}KO}$ mice (line #12) and littermate Atp8b1$^{flox/flox}$ mice, suspended in 100 µL of homogenization buffer [75 mM Tris-HCl, pH 7.5 containing 1 mg/ml BSA (Sigma-Aldrich)], and sonicated. The debris was removed by centrifugation at 1000×g for 5 min, and the supernatant was used as the source of Lpcat. Each reaction mixture contained 2 µg of LysoNBD-PC, 10 µg of 18:1 (n9) oleoyl coenzyme A, and the prepared specimens in 100 µL of homogenization buffer. The reaction tube was kept at room temperature for exactly 10 min, and the reaction was terminated by adding 375 µL of chloroform/methanol (1:2, v/v) and mixing vigorously. Lipids were extracted by the Bligh and Dyer method [5]. The organic phase was transferred to a 1.5-ml tube and dried using a centrifugal concentrator (TOMY SEIKO, Tokyo, Japan). Lipids were redissolved in 40 µL of chloroform and applied to a TLC plate. The running solvent was chloroform/methanol/water (65:25:4, v/v). The fluorescence signal was detected by ImageQuanta LAS 4000 (FUJIFILM), and the intensity was measured by ImageJ software (ver. 1.53c; National Institutes of Health).

Flippase assay

The incorporation of NBD-phospholipids was analyzed by flow cytometry as described previously [6,7]. Forty-eight hours after transfection, the transfected CHO-K1 cells and HEK293T cells were detached from dishes in PBS containing 5 mM EDTA and collected by centrifugation. Eight-week-old Atp8b1$^{Tax\text{-}iIEC\text{-}KO}$ mice and littermate Atp8b1$^{flox/flox}$ mice were treated with 1 mg Tax intraperitoneally daily for 4 days and subjected to IEC preparation. The cells (2×10^6 cells/sample) were washed and equilibrated at 15 °C for 15 min in 500 µL of Hanks' Balanced Salt Solution (HBSS; Thermo Fisher Scientific). An equal volume of 1 µg/mL 18:1-06:0 NBD-PC (Avanti Polar Lipids) or 10 µg/mL 12:0 LysoNBD-PC was added to the cell suspension and incubated at 15 °C. At each time point, 300 µL of cell suspension was collected and mixed with 300 µl of ice-cold HBSS containing 5% fatty acid-free BSA (Sigma-Aldrich) to extract NBD-lipids incorporated into the exoplasmic leaflet of the plasma membrane as well as unincorporated NBD-lipids. Next, 10,000 cells were analyzed with a BD FACSCelesta (BD Bioscience, San Jose, CA) to measure fluorescence of NBD-lipids translocated into the cytoplasmic leaflet of the plasma membrane. The mean of the fluorescence intensities per cell was calculated. Cells positive for propidium iodide (DOJINDO, Kumamoto, Japan) were excluded from the analysis.

Cytotoxicity assay

Ten mg/mL LPC (FUJIFILM Wako Pure Chemical) in ethanol/water (1:1, v/v) and 5 mM edelfosine (Cayman, Ann Arbor, MI) in ethanol were prepared as stock solutions. The transfected CHO-K1 cells and HEK293T cells were seeded on a 96-well plate at a cell density of 50,000 cells/well. After 24 h, the cells were incubated with fresh medium supplemented

with different concentrations of edelfosine or LPC for 24 h. Lactate dehydrogenase release from damaged cells was measured using a Cytotoxicity LDH Assay Kit-WST (DOJINDO), in accordance with the manufacturer's instruction.

Immunoblotting

Specimens were loaded into wells of Mini-PROTEAN TGX Gels (BIO-RAD, Hercules, CA), electrophoresed, and analyzed by immunoblotting as described previously [8]. Fusion Solo7S with FusionCapt17 software (Vilber Lourmat, Collégien, France) was used at high resolution and auto exposure to detect immunoreactivity using the WESTAR C ULTRA 2.0 or the WESTAR SUPERNOVA (Cyanagen, Bologna, Italy).

Supplementary Figures

a

	WT allele (bp)	Flox allele (bp)
L-Flox	3728	2206/1631
R-Flox	2543	1485/1120

b

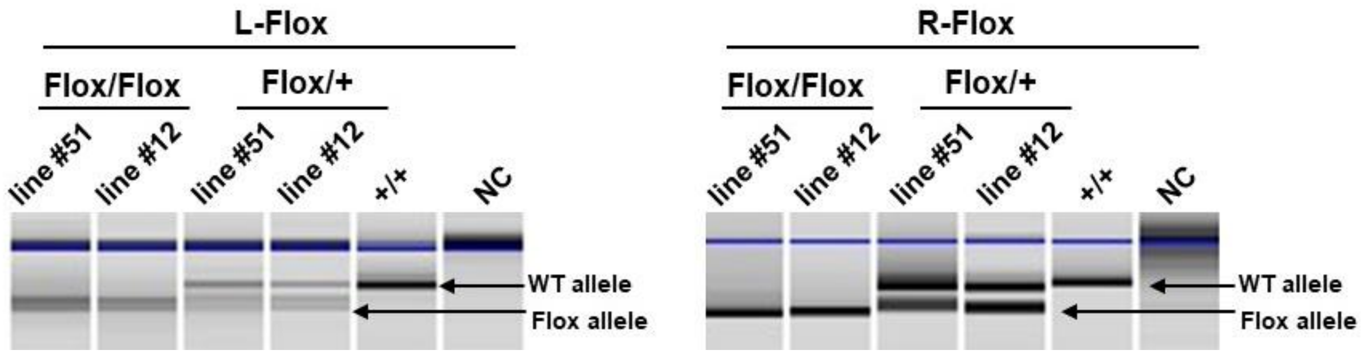

Supplementary Fig. 1. PCR-based genotyping of Atp8b1$^{flox/flox}$ mice, Atp8b1$^{flox/+}$ mice, and Atp8b1$^{+/+}$ mice.

a, Table showing predicted band size of PCR products for genotyping of wild-type allele and flox allele. **b,** Capillary microchip electrophoresis of PCR products. To distinguish wild type allele and flox allele, the genomic DNA was purified from the tail of the pups and subjected to PCR and subsequent reaction with a restriction enzyme, AscI for L-flox and EcoRV for R-flox.

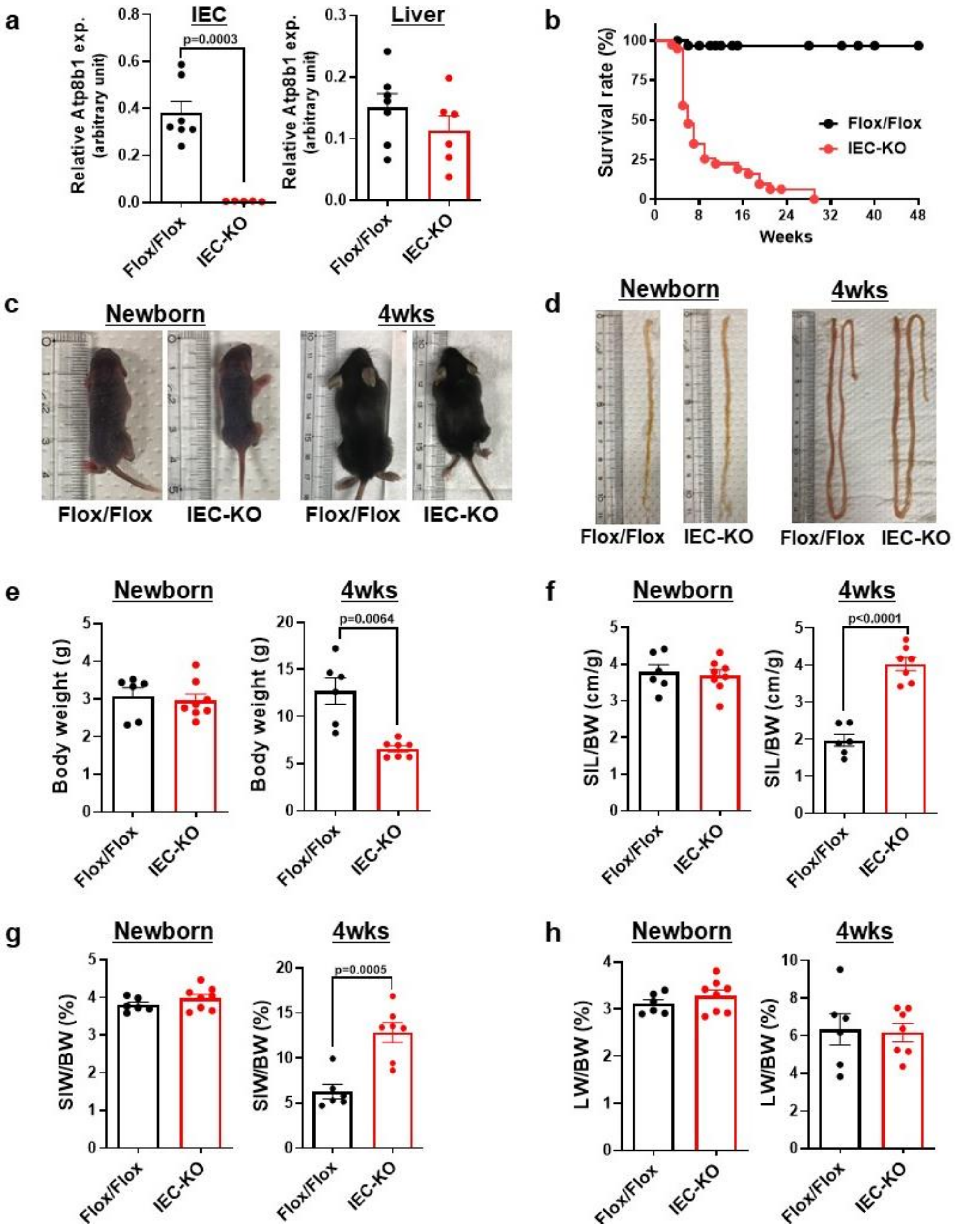

Supplementary Fig. 2. Atp8b1$^{IEC\text{-}KO}$ mice (line #51) show neonatal lethality, growth retardation, and elongation of the SI.

a, Atp8b1 mRNA levels in IEC and liver from male 4-week-old Atp8b1$^{IEC\text{-}KO}$ mice (line #51) (n = 6) and littermate Atp8b1$^{flox/flox}$ mice (n = 7). mRNA levels are expressed relative to those of 18S rRNA. **b,** Survival rate of male Atp8b1$^{IEC\text{-}KO}$ mice (line #51) and littermate Atp8b1$^{flox/flox}$ mice (n = 32–39 in each group). **c–h,** Gross appearance of whole body **(c)** and SI **(d)**, BW **(e)**, SIL to BW **(f),** SIW to BW **(g)**, and LW to BW **(h)** of male newborn and 4-week-old Atp8b1$^{IEC\text{-}KO}$ mice (line #51) (n = 8 for newborn, n = 7 for 4wks) and littermate Atp8b1$^{flox/flox}$ mice (n = 6 in each group). All data are presented as mean ± SEM. *P* values were calculated by two-tailed, unpaired Welch's t-test and indicated in the figures if less than 0.05. BW, body weight; LW, liver weight; SIW, small intestine weight; SIL, small intestine length.

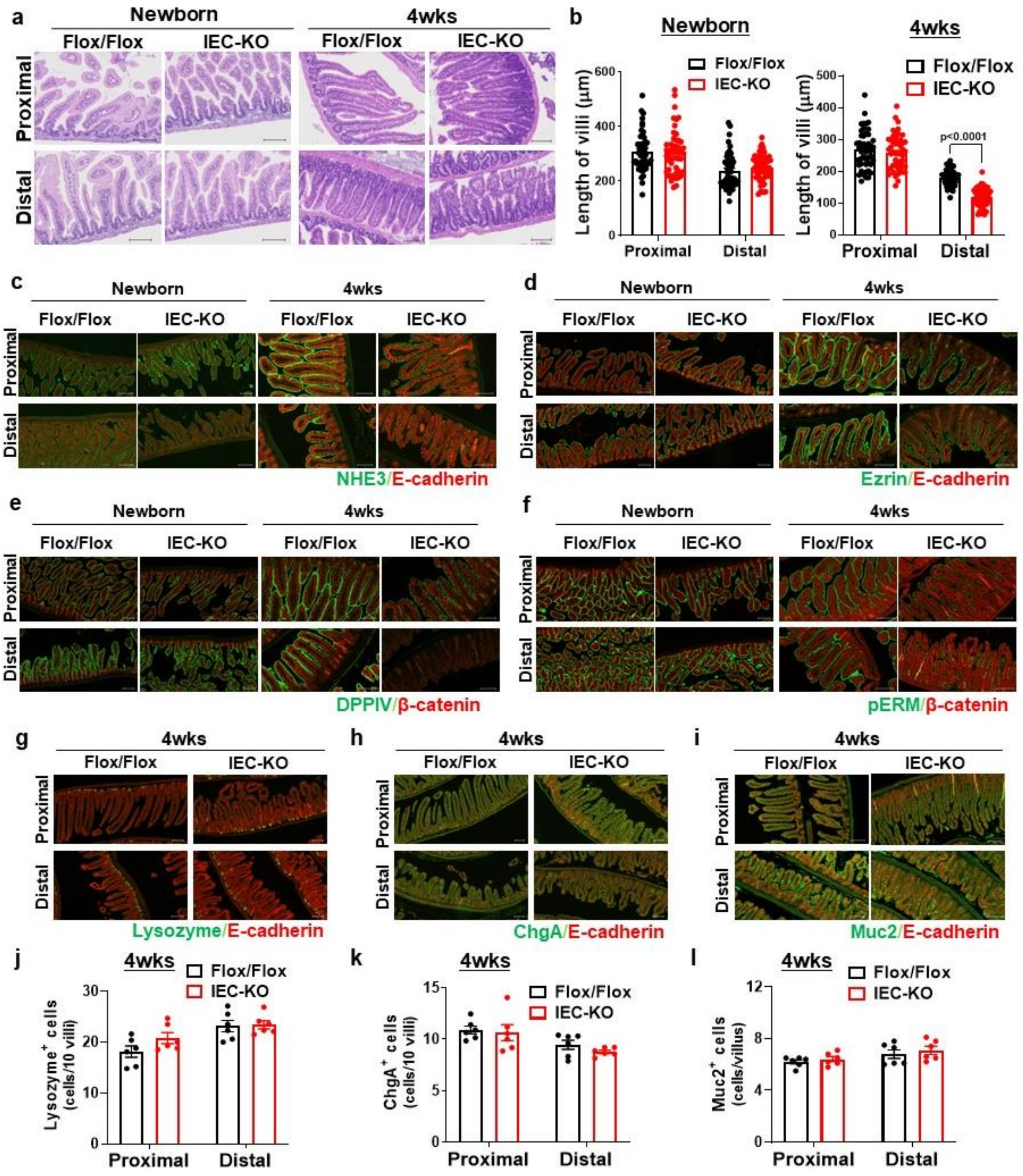

Supplementary Fig. 3. Atp8b1^{IEC-KO} mice (line #51) have shortened villi and lower expression of apical membrane protein in SI. Proximal and distal SI was excised from male Atp8b1^{IEC-KO} mice (line #51) and littermate Atp8b1$^{flox/flox}$ mice at the newborn stage (n = 5) and at 4 week old (n = 6) and subjected to histological analysis. **a,** H&E staining of SI section. **b,** Quantification of villus length. Symbols indicate values of 50 villi in each mouse. **c–f,** IHC staining of apical membrane markers, NHE3 **(c)**, ezrin **(d)**, DPPIV **(e)**, and pERM **(f)**, and basolateral membrane markers, E-cadherin **(c, d)** and β-catenin **(e, f)**, of SI section. **g–i,** IHC staining of IEC markers, lysozyme (Paneth cells; **g**), chgA (endocrine cells; **h**), and muc2 (goblet cells; **i**), in SI sections. **j–l,** Quantification of IEC positive for lysozyme **(j)**, chgA **(k)**, and muc2 **(l)** per villus. In each mouse, more than 50 villi were evaluated from three images. Each symbol indicates values of 6 mice/group. In **a** and **c–i,** representative images are shown. Scale bars: 100 μm. In **b** and **j–l**, all data are presented as mean ± SEM. *P* values were calculated by two-tailed, unpaired Welch's t-test and indicated in the figures if less than 0.05.

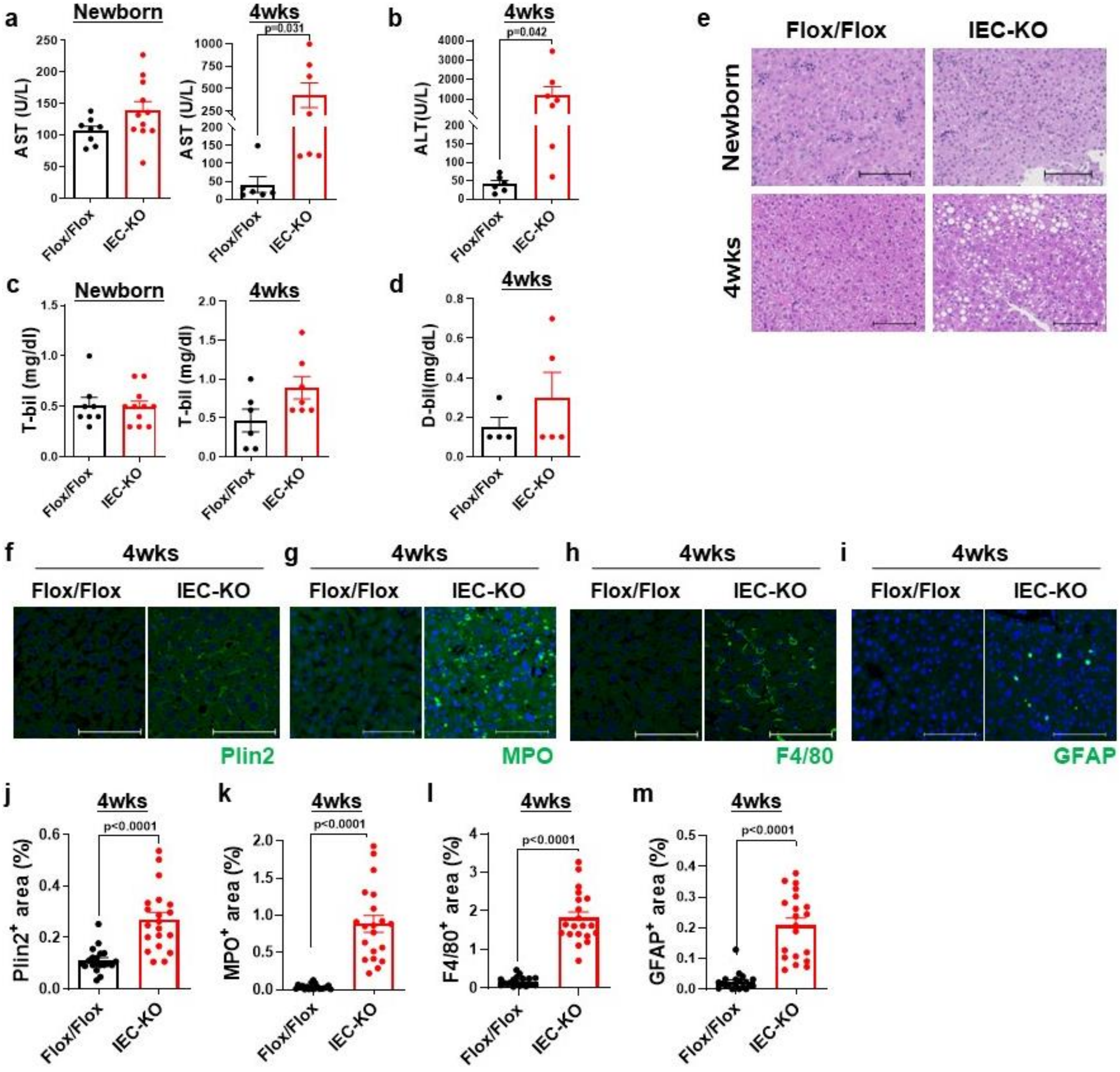

Supplementary Fig. 4. Atp8b1$^{IEC\text{-}KO}$ mice (line #51) progress to steatohepatitis.

Plasma and liver were collected from male Atp8b1$^{IEC\text{-}KO}$ mice (line #51) (n = 11 for newborn, n = 7 for 4wks) and littermate Atp8b1$^{flox/flox}$ mice (n = 8 for newborn, n = 6 for 4wks) at the newborn stage and at 4 weeks old and subjected to biochemical and histological analyses, respectively. **a–d,** Plasma levels of AST **(a)**, ALT **(b),** T-bil **(c)**, and D-bil **(d)**. **e,** HE staining of liver section. **f–i,** IHC staining of liver section by antibodies against Plin2 (lipid droplets; **f),** MPO (neutrophils; **g**), F4/80 (macrophages; **h**), and GFAP (quiescent HSC; **i**). **j–m,** Quantification of the area stained with Plin2 **(j)**, MPO **(k)**, F4/80 **(l)**, and GFAP **(m)**. Each symbol indicates values of 20 images/group from 5 mice/group. In **e–i**, representative images are shown. Scale bars: 100 µm. In **a–d** and **j–m**, all data are presented as mean ± SEM. *P* values were calculated by two-tailed, unpaired Welch's t-test and indicated in the figures if less than 0.05.

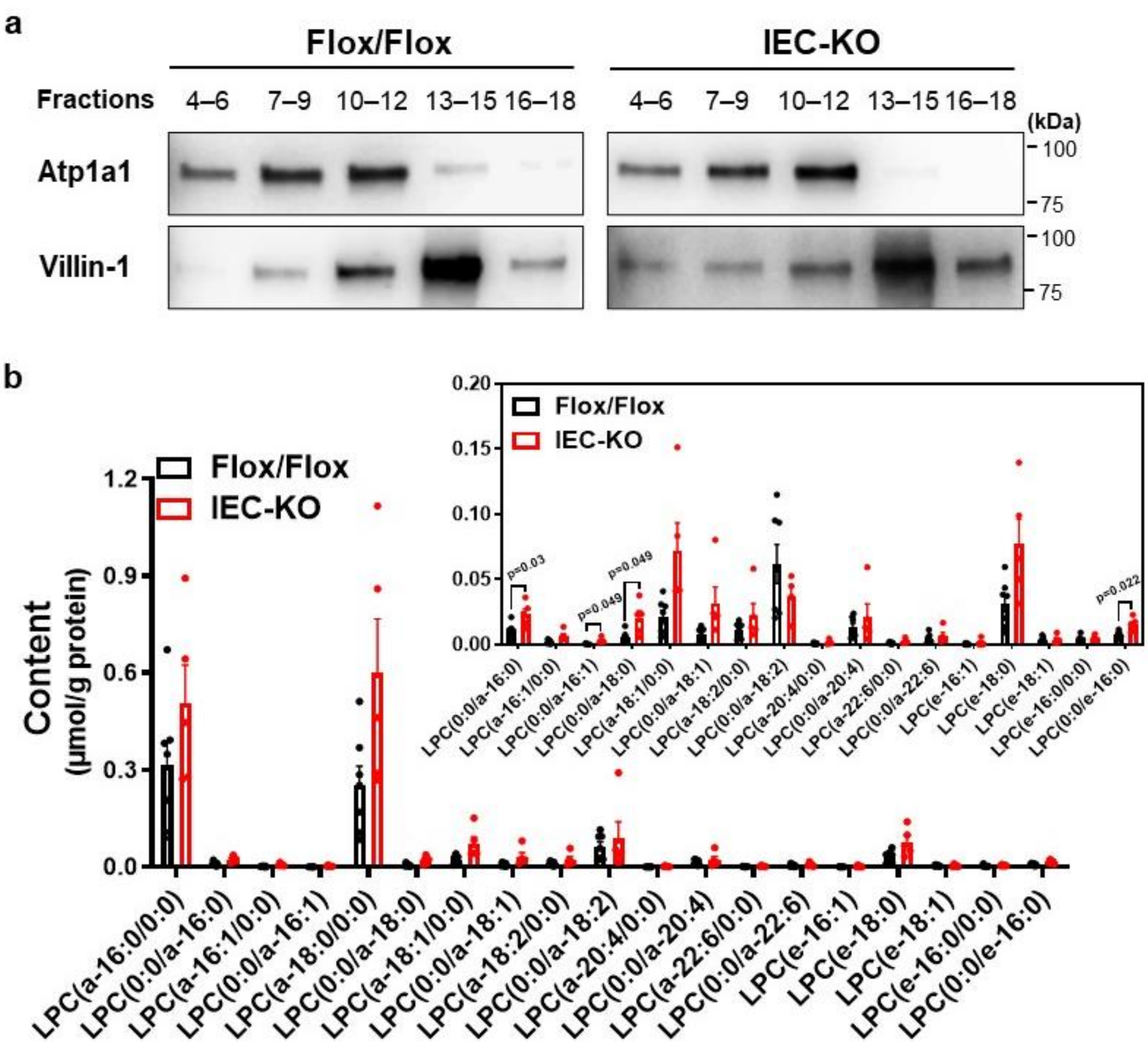

Supplementary Fig. 5. Atp8b1$^{IEC\text{-}KO}$ mice (line #12) show LPC accumulation in the brush border membranes of IEC. Male four-week-old Atp8b1$^{IEC\text{-}KO}$ mice (line #12) (n = 5) and littermate Atp8b1$^{flox/flox}$ mice (n = 7) were subjected to the preparation of the brush border membrane of IEC. The prepared specimens were investigated by immunoblotting **(a)** and targeted lipidomic analysis of lysophospholipids **(b)**. **a,** Abundance of Atp1a1 and villin, a marker of basolateral membrane and brush border membranes, respectively, in fractions of iodixanol density-gradient of IEC membranes. The prepared specimens were pooled for each of the three fractions (4–6, 7–9, 10–12, 13–15, and 16–18) and subjected to immunoblotting. Representative images of two independent experiments are shown. **b,** The levels of the indicated LPC species in the brush border membrane of IEC. Fractions 13–15 (the brush border membranes of IEC) were pooled and subjected to the targeted lipidomic analysis. Data are presented as mean ± SEM. *P* values were calculated by two-tailed, unpaired Welch's t-test and indicated in the figures if less than 0.05.

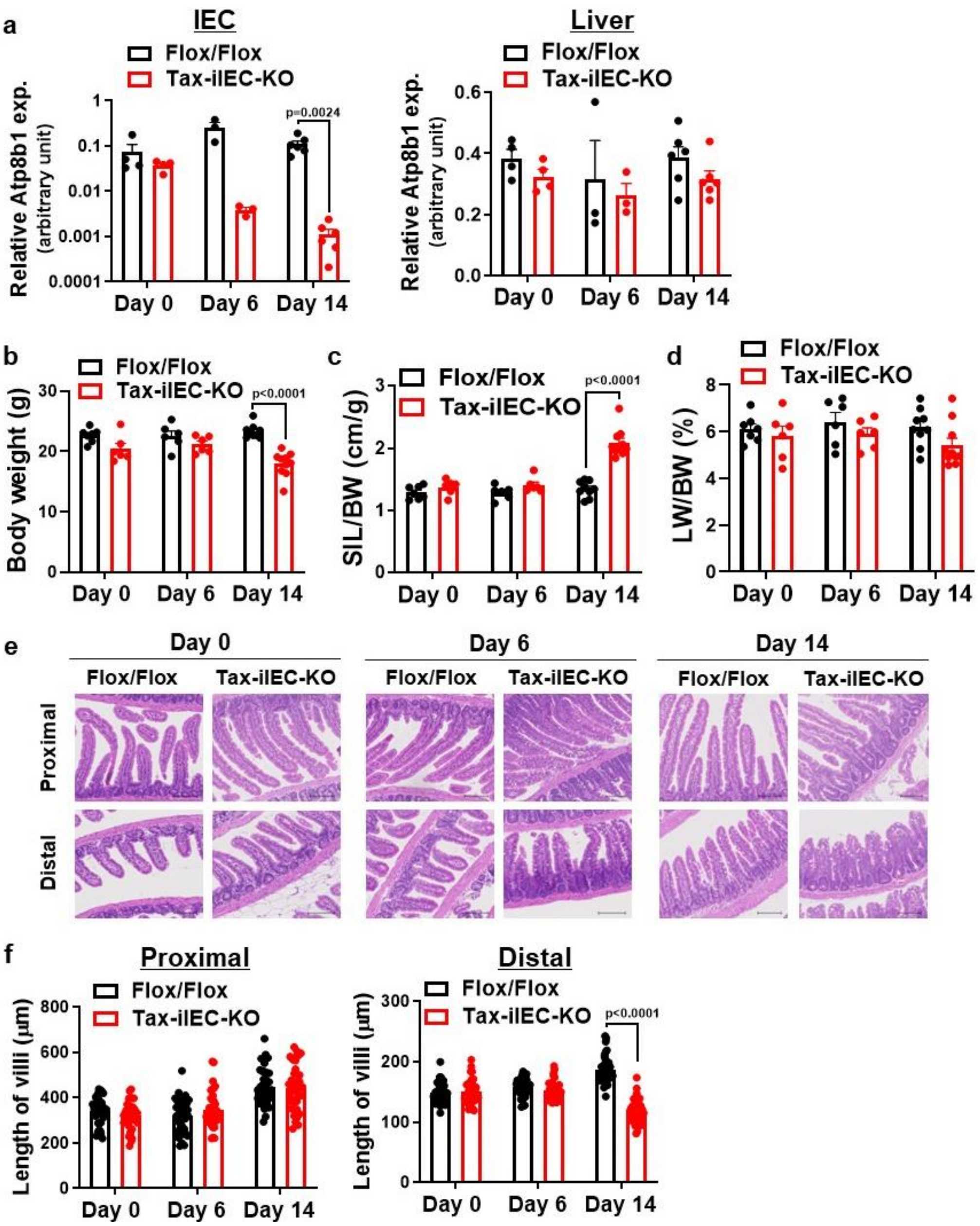

Supplementary Fig. 6. Weight loss and intestinal abnormality in Atp8b1$^{\text{Tax-iIEC-KO}}$ mice (line #12) after Tax treatment.

Male 8-week-old Atp8b1$^{\text{Tax-iIEC-KO}}$ mice (line #12) and littermate Atp8b1$^{\text{flox/flox}}$ mice were treated daily with or without 1 mg Tax intraperitoneally for 4 days and then subjected to collect liver and SI on day 6 or 14 after the start of Tax administration. **a,** Atp8b1 mRNA levels in IEC and liver (n = 4, 3, and 6 for day 0, 6, and 14). mRNA levels are expressed relative to those of 18S rRNA. **b–d,** BW **(b)**, SIL to BW **(c),** and LW to BW **(d)** [n = 7, 6, and 9 (Atp8b1$^{\text{flox/flox}}$) and 6, 6, and 10 (Atp8b1$^{\text{Tax-iIEC-KO}}$) for day 0, 6, and 14]. **e,** H&E staining of SI section. Representative images are shown. Scale bars: 100 μm. **f,** Quantification of villus length. Symbols indicate values of 40 villi from 5 mice in each group. In **a–d** and **f**, all data are presented as mean ± SEM. *P* values were calculated by two-tailed, unpaired Welch's t-test and indicated in the figures if less than 0.05. BW, body weight; LW, liver weight; SIL, small intestine length.

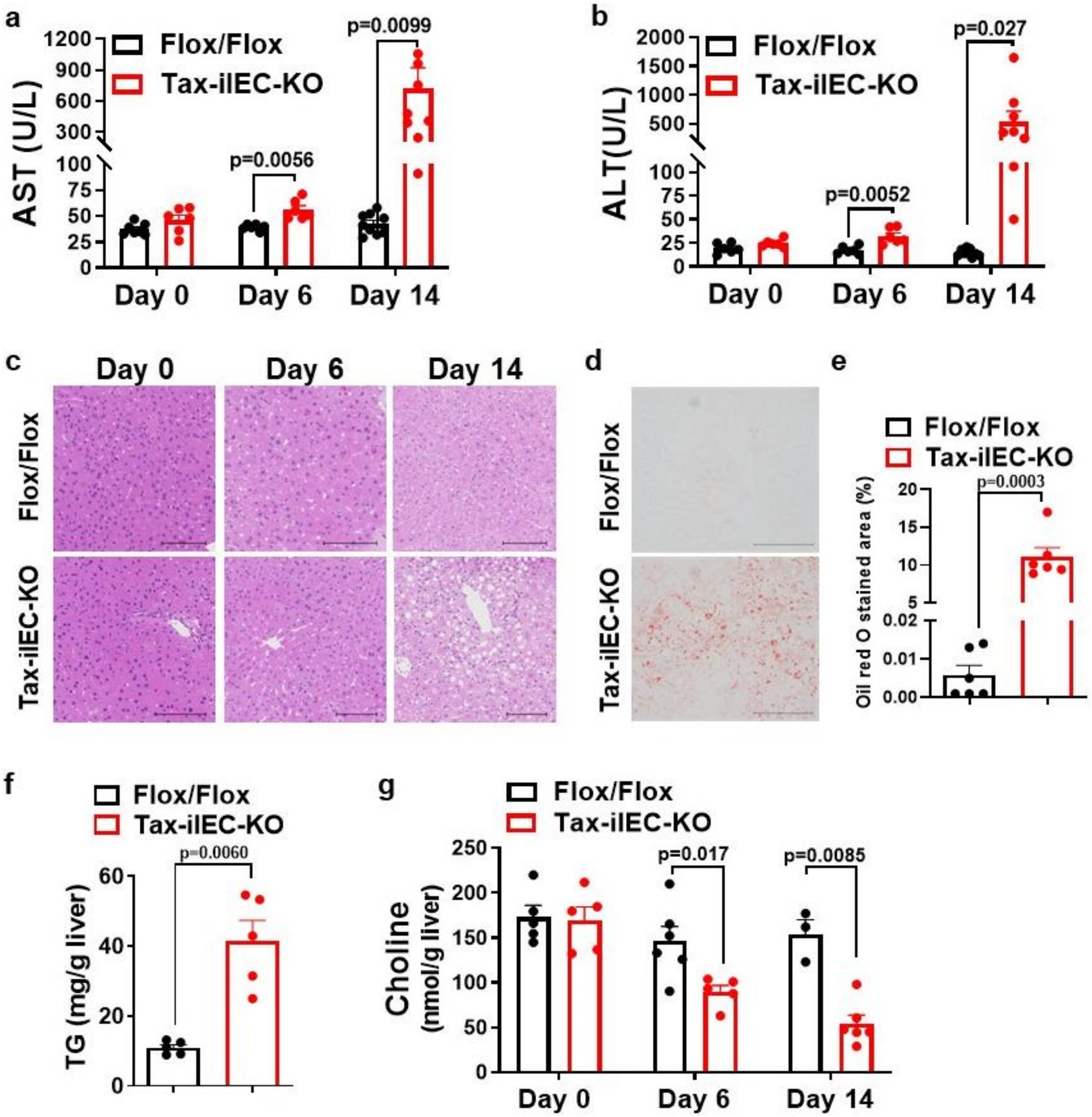

Supplementary Fig. 7. Decreased liver choline level precedes weight loss and intestinal abnormality in Atp8b1$^{Tax\text{-}iIEC\text{-}KO}$ mice (line #12) after Tax treatment.

Male 8-week-old Atp8b1$^{Tax\text{-}iIEC\text{-}KO}$ mice (line #12) and littermate Atp8b1$^{flox/flox}$ mice were treated daily with or without 1 mg Tax intraperitoneally for 4 days and then subjected to collect blood and liver on day 6 or 14 after the start of Tax administration. **a–b,** Plasma levels of AST **(a)**, ALT **(b)** (n = 6, 6, and 9 for day 0, 6, and 14)**. c–d,** H&E staining **(c)** and Oil red O staining **(d)** of the liver section. Representative images are shown. Scale bars: 100 µm. **e,** Quantification of Oil red O-stained area. Each symbol indicates 6 images from 3 mice in each group. **f,** Enzymatic determination of total TG in the liver (n = 5). **g,** Liver choline level [n = 5, 6, and 3 (Atp8b1$^{flox/flox}$) and 5, 5, and 6 (Atp8b1$^{Tax\text{-}iIEC\text{-}KO}$) for day 0, 6, and 14]. In **a, b, e–g**, all data are presented as mean ± SEM. *P* values were calculated by two-tailed, unpaired Welch's t-test and indicated in the figures if less than 0.05.

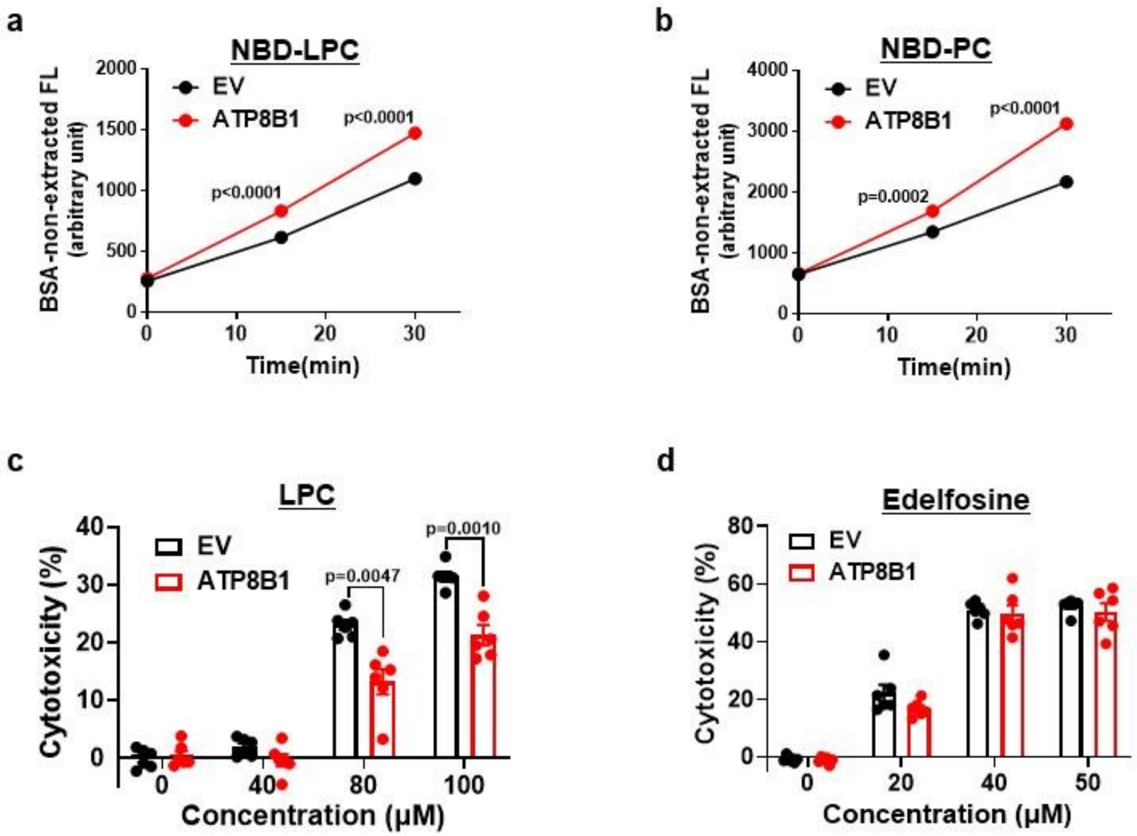

Supplementary Fig. 8. Atp8b1 has flipping activity to LPC in HEK293T cells.

HEK293T cells were transfected with pShuttle-ATP8B1–FLAG or corresponding empty vector and analyzed to evaluate flippase activity to NBD-LPC **(a)** and NBD-PC **(b)** and susceptibility to toxicity of LPC **(c)** and edelfosine **(d)**, a synthetic lipase-resistant LPC analog. As described in Supplementary materials, for flippase activity measurement **(a, b)**, the cells incubated with NBD-lipids were washed with 5% fatty acid-free BSA to remove NBD-lipids incorporated into the exoplasmic leaflet of the plasma membrane and then analyzed by FACS. Each bar represents the mean ± SEM of quadruple **(a, b)** or sextuple **(c, d)** determinations. A representative result of two independent experiments is shown. *P* values were calculated by two-tailed, unpaired Welch's t-test and indicated in the figures if less than 0.05. EV, empty vector.

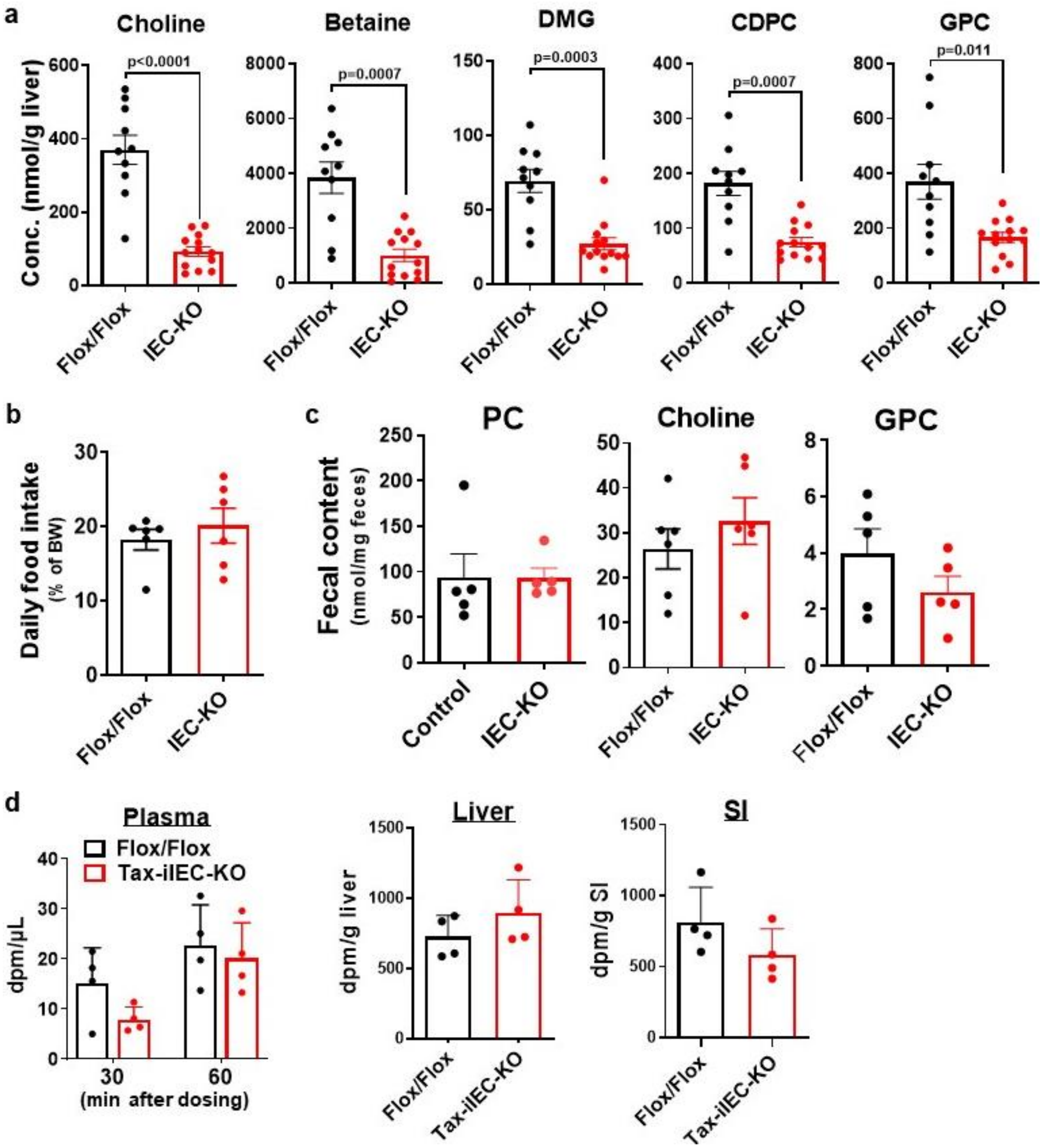

Supplementary Fig. 9. Atp8b1^{IEC-KO} mice (line #12) has low levels of hepatic choline metabolites but normal food intake and intestinal choline absorption.

Male 4-week-old Atp8b1^{IEC-KO} mice (line #12) [n = 13 **(a)**, n = 6 **(b–d)**] and littermate Atp8b1$^{flox/flox}$ mice [n = 10 **(a)**, n = 6 **(b–d)**] were analyzed to evaluate hepatic choline metabolites **(a)**, food intake **(b)**, and fecal content of PC, GPC, and choline **(c)**. Male 8-week-old Atp8b1$^{Tax-iIEC-KO}$ mice (line #12) and littermate Atp8b1$^{flox/flox}$ mice were treated daily with 1mg of TAX intraperitoneally for 5 days and then orally administered a choline chloride solution (50 mg/kg body weight) containing [methyl-^{3}H]-choline chloride (150 μCi/kg body weight) (n = 4) **(d)**. Plasma, liver, and SI were collected as described in Supplementary methods, and [^{3}H]-radioactivity in these specimens was evaluated. Data are presented as mean ± SEM. *P* values were calculated by two-tailed, unpaired Welch's t-test and indicated in the figures if less than 0.05. CDPC, CDP-choline; DMG, dimethylglycine, GPC, glycerophosphorylcholine.

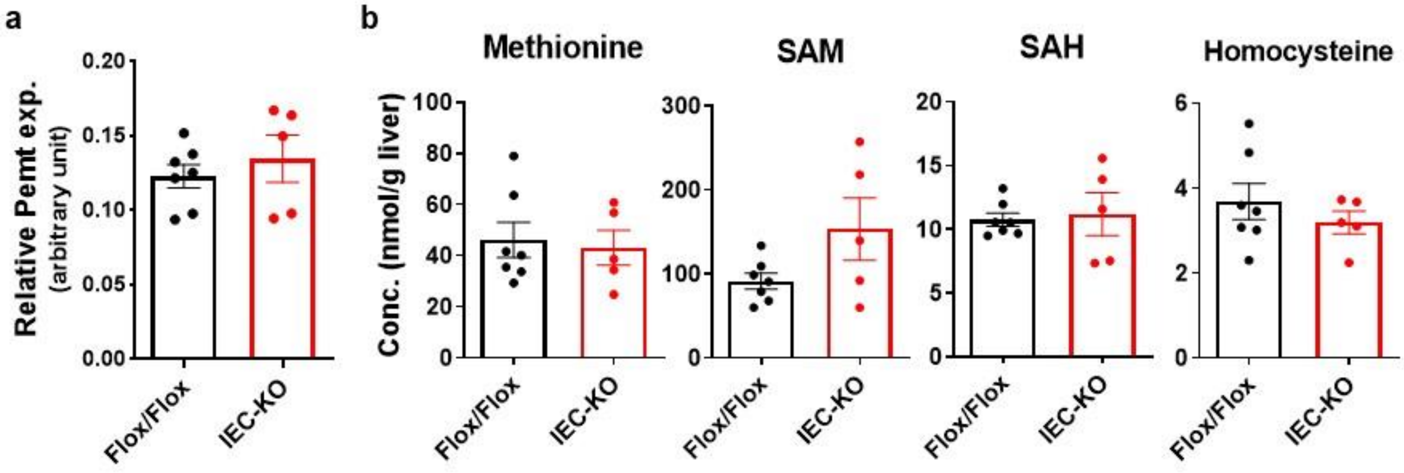

Supplementary Fig. 10. Atp8b1$^{IEC\text{-}KO}$ mice (line #12) has levels of hepatic Pemt mRNA and methionine metabolites comparable to the littermate Atp8b1$^{flox/flox}$ mice.

Liver was collected from male Atp8b1$^{IEC\text{-}KO}$ mice (line #12) (n = 5) and littermate Atp8b1$^{flox/flox}$ mice (n = 7) at 4 weeks old and analyzed to evaluate Pemt mRNA **(a)** and methionine metabolites **(b)**. Data are presented as mean ± SEM. In **a**, mRNA levels are expressed relative to those of 18S rRNA. Pemt, phosphatidylethanolamine N-methyltransferase; SAH, S-adenosyl-homocysteine; SAM, S-Adenosyl-methionine.

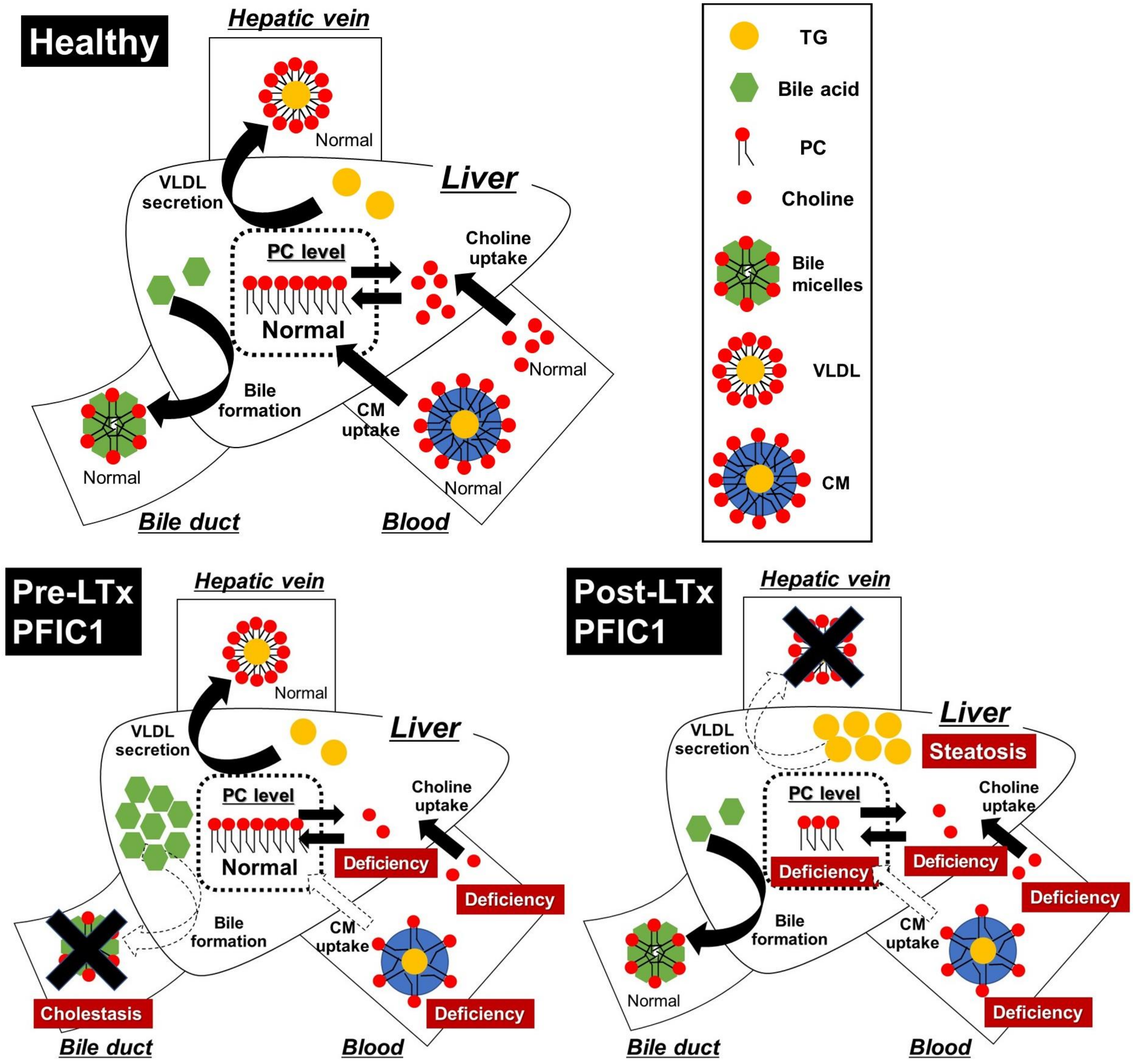

Supplementary Fig. 11. Schematic diagram illustrating hepatic PC levels in healthy individuals and PFIC1 patients. In both pre-LTx and post-LTx PFIC1 patients, PC supply to the liver through hepatic uptake of CM is reduced because of poor LPC absorption in IEC, resulting in hepatic choline deficiency. Pre-LTx PFIC1 patients suffer from severe cholestasis, a disease state characterized by impaired bile formation, which allows bile constituents including PC to accumulate in the liver and prevent hepatic PC deficiency. Therefore, only in post-LTx PFIC1 patients is the hepatic PC level below the lower limit able to maintain normal VLDL secretion, leading to the development of steatosis.

Supplementary Tables

Supplementary Table 1. Mendelian distribution of newborn mice from mating Atp8b1$^{flox/+}$;villin-Cre mice with Atp8b1$^{flox/flox}$ mice

**Genotype**	**Expected %**	**Observed no. (%) line #51**	**Observed no. (%) line #12**
Flox/Flox	25%	26 (26.5%)	16 (19.0%)
Flox/Flox, Villin-Cre	25%	20 (20.4%)	22 (26.2%)
Flox/+	25%	24 (24.5%)	19 (22.6%)
Flox/+, Villin-Cre	25%	28 (28.6%)	27 (32.1%)
Total	100%	98 (100%)	84 (100%)

Supplementary Table 2. Detailed information of patients with other cholestasis in Table 1

Other cholestasis pre-LTx (n=27)		Other cholestasis post-LTx (n=20)	
**Disease name**	**No. of patients**	**Disease name**	**No. of patients**
PFIC2	8	PFIC2	14
PFIC3	1	PFIC3	1
PFIC4	1	PFIC5	1
PFIC5	1	Normal GGT cholestasis without genetic diagnosis	4
BRIC2	2		
Normal GGT cholestasis without genetic diagnosis	14		

Supplementary Table 3. Degree of hepatic steatosis and diarrhea and plasma choline levels in PFIC1 patients in Table 1

	Case no.	Years after LTx	Steatosis (histological assessment)	Diarrhea	Plasma choline (µM)
PFIC1 pre-LTx	1	NA	–	+	11.2
	2	NA	–	–	10.4
	3	NA	–	+	9.6
	4	NA	–	+	7.5
	5	NA	–	+	14.5
	6	NA	–	–	21.4
	7	NA	–	–	8.4
	8	NA	–	+	10.8
	9	NA	–	–	9
	10	NA	–	–	17.6
PFIC1 post-LTx	1	0.5	Macrovesicular steatosis, 60%	–	5.4
	2	0.6	Macrovesicular steatosis, 66%	NA (intestinal stoma)	4.9
	3	0.6	Steatohepatitis, Mixed steatosis, 80%	–	6.4
	4	1.5	Steatohepatitis, Mixed steatosis 70%	–	3.9
	5	12.2	Steatohepatitis, Macrovesicular steatosis, 60%	+	8.1
	6	13.6	Steatohepatitis, Mixed steatosis, 60%	+	2.2
	7	8.5	Steatohepatitis, Mixed steatosis, 50%	+	3.9
	8	9.0	Steatohepatitis, Mixed steatosis, 50%	+	4.6
	9	22.1	Steatohepatitis, Mixed steatosis, 50%	+	3.7
	10	3.9	Steatohepatitis, Mixed steatosis, 40%	+	5.1
	11	5.8	Macrovesicular steatosis, 60%	–	8.5
	12	12.3	Macrosteatosis, 30%	–	12.8

LTx, liver transplantation; NA, not applicable.

Supplementary Table 4. Reagents used in this study

Reagent	Source	Identifier
Ethanol 99.5%	FUJIFILM Wako Pure Chemical, Osaka, Japan	055-00457
Xylene	FUJIFILM Wako Pure Chemical, Osaka, Japan	242-00087
pure Eosin	MUTO Pure Chemical, Tokyo, Japan	32042
Hematoxylin	Merck, Branchburg, NJ	1.04302.0025
Entellan new	Merck, Branchburg, NJ	107961
KOD One	TOYOBO, Osaka, Japan	KMM-101
KOD-Fx	TOYOBO, Osaka, Japan	KFX-101
ReverTra Ace® qPCR RT Master Mix with gDNA Remover	TOYOBO, Osaka, Japan	FSQ-301
THUNDERBIRD SYBR qPCR Mix	TOYOBO, Osaka, Japan	QPS-201
10% Formalin Neutral Buffer Solution	FUJIFILM Wako Pure Chemical, Osaka, Japan	062-01661
Dulbecco’s PBS(－)	Nissui, Tokyo, Japan	5913
Taurocholic acid sodium salt hydrate 97%	Sigma-Aldrich, St. Louis, MO	861960
TrypLE express Enzyme(1×), no phenol red	Thermo fisher scientific, Waltham, MA	12604021
2NA(EDTA・2Na)	Dojindo, Kumamoto, Japan	345-01865
ISOGEN II	Nippon gene, Tokyo, Japan	311-07361
RNeasy Mini Kit (50)	QIAGEN, Hilden, Germany	74104
FastGene Plasmid mini Kit	Nippon genetics, Tokyo, Japan	FG-90502
FDC GPT/ALT-P3 50 slides	FUJIFILM, Tokyo, Japan	15211733
FDC TBIL-P3 50 slides	FUJIFILM, Tokyo, Japan	15514563
LabAssay Triglyceride	FUJIFILM, Tokyo, Japan	632-50991
LabAssay Phospholipid	FUJIFILM, Tokyo, Japan	639-51001
BD Horizon™ Dri Tumor & Tissue Dissociation Reagent	BD, New Jersey, US	661563
DMEM, high glucose, pyruvate	Thermo Fisher Scientific, San Jose, CA	11995073
Tris-HCl Buffer, pH 10, 10×, Antigen Retriever	Sigma-Aldrich, St. Louis, MO	T6455-1000ML
18:1 (n9) oleoyl coenzyme A	Avanti Polar Lipids, Alabaster, AL	870719P
12:0 LysoNBD-PC	Avanti Polar Lipids, Alabaster, AL	810128P
18:1-06:0 NBD-PC	Avanti Polar Lipids, Alabaster, AL	810132
Bovine serum albumin	Sigma-Aldrich, St. Louis, MO	A6003
HBSS	Thermo Fisher Scientific, San Jose, CA	14025092
DMEM/F12 (1:1)	Thermo Fisher Scientific, San Jose, CA	11320
Fetal bovine serum	Thermo Fisher Scientific, San Jose, CA	10437-028
Propidium iodide	DOJINDO, Kumamoto, Japan	P378
Penicillin-Streptomycin Solution	FUJIFILM Wako Pure Chemical, Osaka, Japan	168-23191
Lysophosphatidylcholine	FUJIFILM Wako Pure Chemical, Osaka, Japan	123-03781
Edelfosine	Cayman, Ann Arbor, MI	CAY-60912-10
Cytotoxicity LDH Assay Kit-WST	DOJINDO, Kumamoto, Japan	CK12
Tamoxifen	Sigma-Aldrich, St. Louis, MO	T5648
Corn oil	FUJIFILM Wako Pure Chemical	032-17016
Choline Chloride	Tokyo Chemical Industry, Tokyo, Japan	C0329-25G
CHOLINE CHLORIDE, [METHYL-3H]-	PerkinElmer, Waltham, MA	NET109
SOLVABLE	PerkinElmer, Waltham, MA	6NE9100
Phosphocholine Chloride Calcium Salt Tetrahydrate	Tokyo Chemical Industry, Tokyo, Japan	P0274-25G
sn-Glycero-3-phosphocholine	Bachem, Bubendorf, Switzerland	4030680.003
Cytidine 5'-Diphosphocholine	FUJIFILM Wako Pure Chemical, Osaka, Japan	032-14071
Betaine	FUJIFILM Wako Pure Chemical, Osaka, Japan	023-10862
N,N-Dimethylglycine	FUJIFILM Wako Pure Chemical, Osaka, Japan	350-30981
Choline-d9 Chloride	Toronto Research Chemicals, Toronto, Canada	C432652
Betaine-13C5,15N, 98 atom %15N, 99 atom %13C, 97% (CP)	Sigma-Aldrich, St. Louis, MO	792322
N-Iodoacetyltyramine	Toronto Research Chemicals, Toronto, Canada	I685880
DL-Homocysteine (3,3,4,4-D4, 98%)	Cambridge isotope laboratories, Andover, MA	DLM-8259
Heptafluorobutyric acid	Tokyo Chemical Industry, Tokyo, Japan	A5713
Methanol -Plus-	KANTO CHEMICAL, Tokyo, Japan	25185-76
Acetonitrile	FUJIFILM Wako Pure Chemical, Osaka, Japan	018-19853
Distilled water -Plus-	KANTO CHEMICAL, Tokyo, Japan	11307-76
Ammonium Acetate	FUJIFILM Wako Pure Chemical, Osaka, Japan	015-02832
OptiPrep	Serumwerk, Bernburg, Germany	1893
Sodium Borofluoride	Nacalai tesque, Kyoto, Japan	31420-82
Hepes	Sigma-Aldrich, St. Louis, MO	H4034
NaCl	FUJIFILM Wako Pure Chemical, Osaka, Japan	191-01665
1,4-Dithiothreitol	Sigma-Aldrich, St. Louis, MO	DTT-RO
EGTA	Dojindo, Kumamoto, Japan	342-01314
$CaCl_2$	FUJIFILM Wako Pure Chemical, Osaka, Japan	038-24985
Trizma® base	Sigma-Aldrich, St. Louis, MO	T1503

Supplementary Table 5. List of primers for qPCR

	GenBank accession no.	Forward primer (5’ →3’)	Reverse primer (5’ →3’)
**Atp8b1**	NM_001001488.3	CGGTTGAACCAGAACAGAATCG	TGGAACTTTCGGTCATTTGCTTT
**Pemt**	NM_008819.3	GAGTGACCACATTTCCCTTCAG	GGAGAGCAACCACGTAGACAA
**18S rRNA**	X56974.1	GTAACCCGTTGAACCCCATT	CCATCCAATCGGTAGTAGCG

Supplementary Table 6. Antibodies used in this study

Antibodies	Manufacture	Identifier	Clone	Application	Dilution
Rat anti-F4/80	Biolegend, San Diego, CA	123101	BM8	IHC	1:100
Goat anti-GFAP	Abcam, Cambridge, UK	ab53554	polyclonal	IHC	1:100
Rabbit anti-αSMA	Cell Signaling Technology	#19245	D4K9N	IHC	1:100
Mouse anti-Plin2	PROGEN, Heidelberg, Germany	610102	AP125	IHC	1:100
Rabbit anti-MPO	Dako, Santa Clara, CA	A0398	polyclonal	IHC	1:100
Rabbit anti-Lysozyme	Abcam, Cambridge, UK	ab108508	polyclonal	IHC	1:200
Rabbit anti-Chga	Abcam, Cambridge, UK	ab15160	polyclonal	IHC	1:200
Rabbit anti-Muc2	Novus Biologicals, Centennial, CO	NBP1-31231	polyclonal	IHC	1:200
Rabbit anti-NHE3	Novus Biologicals, Centennial, CO	NBP1-82574	polyclonal	IHC	1:200
Goat anti-DPPIV	R&D Systems, Minneapolis, MN	AF954	polyclonal	IHC	1:200
Rabbit anti-Ezrin	Cell Signaling Technology, Danvers, MA	3145	polyclonal	IHC	1:200
Rabbit anti-pERM	Cell Signaling Technology, Danvers, MA	3726	48G2	IHC	1:200
Mouse anti-E-cadherin	BD Bioscience, San Jose, CA	610182	36	IHC	1:200
Rabbit anti-β-catenin	Cell Signaling Technology, Danvers, MA	19807	D2U8Y	IHC	1:200
Mouse anti-ATP1A1	Santa Cruz Biotechnology, Heidelberg, Germany	sc-21712	C464.6	IB	1:200
Rabbit anti-Villin-1	Cell Signaling Technology, Danvers, MA	2369	polyclonal	IHC/IB	1:100/1:2000
Alexa Fluor 488 donkey anti-goat IgG	Thermo Fisher Scientific, San Jose, CA	A11055	polyclonal	IHC	1:250
Alexa Fluor 488 donkey anti-mouse IgG	Thermo Fisher Scientific, San Jose, CA	A21202	polyclonal	IHC	1:250
Alexa Fluor 546 donkey anti-mouse IgG	Thermo Fisher Scientific, San Jose, CA	A10036	polyclonal	IHC	1:250
Alexa Fluor 488 goat anti-rabbit IgG	Thermo Fisher Scientific, San Jose, CA	A11008	polyclonal	IHC	1:250
Alexa Fluor 546 donkey anti-rabbit IgG	Thermo Fisher Scientific, San Jose, CA	A10040	polyclonal	IHC	1:250
Alexa Fluor 488 donkey anti-rat IgG	Thermo Fisher Scientific, San Jose, CA	A21208	polyclonal	IHC	1:250

IHC, immunohistochemistry; IB, immunoblotting.

Supplementary Table 7. Selected reaction monitoring parameters for the LC/MS/MS assay to measure choline metabolites

Name	Q1	Q3	Dwell time	DP	EP	CE	CXP
Choline	104	60	100	90	10	21	10
Betaine	118	59	100	90	10	41	10
N,N-dimethylglycine	104	58	100	70	10	21	10
Choline-IS	113	69	100	90	10	21	10
Betaine-IS	124	62	100	90	10	51	10

DP: declustering potential (volts), EP: entrance potential (volts), CE: collision energy (volts), CXP: collision cell exit potential (volts), Dwell time (msec.)

Supplementary Table 8. Selected reaction monitoring parameters for the LC/MS/MS assay to measure methionine metabolites

Name	Q1	Q3	Dwell time	DP	EP	CE	CXP
Methionine	150.1	133	75	90	10	12	10
S-adenosyl homocysteine	385	136	75	81	10	27	10
S-adenosyl methionine	399.1	250	75	90	10	15	10
Homocysteine-HPEAM	313.1	121.1	75	40	10	45	10
Homocysteine-HPEAM-IS	317.1	121.1	75	40	10	45	10

DP: declustering potential (volts), EP: entrance potential (volts), CE: collision energy (volts), CXP: collision cell exit potential (volts), Dwell time (msec.)

Supplementary references

1. Yuan, M., Breitkopf, S. B., Yang, X. & Asara, J. M. A positive/negative ion-switching, targeted mass spectrometry-based metabolomics platform for bodily fluids, cells, and fresh and fixed tissue. *Nat. Protoc.* **7**, 872–881 (2012).

2. Satomi, Y., Hirayama, M. & Kobayashi, H. One-step lipid extraction for plasma lipidomics analysis by liquid chromatography mass spectrometry. *J. Chromatogr. B Analyt. Technol. Biomed. Life Sci.* **1063**, 93–100 (2017).

3. Takeda, H. *et al.* Widely-targeted quantitative lipidomics method by supercritical fluid chromatography triple quadrupole mass spectrometry. *J. Lipid Res.* **59**, 1283–1293 (2018).

4. Li, Z. *et al.* Lysophosphatidylcholine acyltransferase 3 knockdown-mediated liver lysophosphatidylcholine accumulation promotes very low density lipoprotein production by enhancing microsomal triglyceride transfer protein expression. *J. Biol. Chem.* **287**, 20122–20131 (2012).

5. Bligh, E. G. & Dyer, W. J. A rapid method of total lipid extraction and purification. *Can. J. Biochem. Physiol.* **37**, 911–917 (1959).

6. Takatsu, H. *et al.* Phospholipid flippase activities and substrate specificities of human type IV P-type ATPases localized to the plasma membrane. *J. Biol. Chem.* **289**, 33543–33556 (2014).

7. Hayashi, H. *et al.* Assessment of ATP8B1 Deficiency in Pediatric Patients With Cholestasis Using Peripheral Blood Monocyte-Derived Macrophages. *EBioMedicine* **27**, 187–199 (2018).

8. Hayashi, H. & Sugiyama, Y. 4-phenylbutyrate enhances the cell surface expression and the transport capacity of wild-type and mutated bile salt export pumps. *Hepatology* **45**, 1506–1516 (2007).
