# Supplementary material for: Intestinal Atp8b1 dysfunction causes hepatic choline deficiency and steatohepatitis
Source: Nat Commun. 2023 Nov 21;14:6763. doi: 10.1038/s41467-023-42424-x (PMC10663612; doi:10.1038/s41467-023-42424-x)
Supplement: Supplementary file 3 — Reporting Summary [file 41467_2023_42424_MOESM3_ESM.pdf]

nature portfolio

Corresponding author(s): Hisamitsu Hayashi

Last updated by author(s): Sep 14, 2023

Reporting Summary

Nature Portfolio wishes to improve the reproducibility of the work that we publish. This form provides structure for consistency and transparency in reporting. For further information on Nature Portfolio policies, see our Editorial Policies and the Editorial Policy Checklist.

Statistics

For all statistical analyses, confirm that the following items are present in the figure legend, table legend, main text, or Methods section.

n/a	Confirmed	
☐	☒	The exact sample size (*n*) for each experimental group/condition, given as a discrete number and unit of measurement
☐	☒	A statement on whether measurements were taken from distinct samples or whether the same sample was measured repeatedly
☐	☒	The statistical test(s) used AND whether they are one- or two-sided<br>*Only common tests should be described solely by name; describe more complex techniques in the Methods section.*
☒	☐	A description of all covariates tested
☐	☒	A description of any assumptions or corrections, such as tests of normality and adjustment for multiple comparisons
☐	☒	A full description of the statistical parameters including central tendency (e.g. means) or other basic estimates (e.g. regression coefficient) AND variation (e.g. standard deviation) or associated estimates of uncertainty (e.g. confidence intervals)
☐	☒	For null hypothesis testing, the test statistic (e.g. *F*, *t*, *r*) with confidence intervals, effect sizes, degrees of freedom and *P* value noted<br>*Give P values as exact values whenever suitable.*
☒	☐	For Bayesian analysis, information on the choice of priors and Markov chain Monte Carlo settings
☒	☐	For hierarchical and complex designs, identification of the appropriate level for tests and full reporting of outcomes
☒	☐	Estimates of effect sizes (e.g. Cohen's *d*, Pearson's *r*), indicating how they were calculated

Our web collection on statistics for biologists contains articles on many of the points above.

Software and code

Policy information about availability of computer code

Data collection	[qPCR]: CFX Connect Real-Time System (Bio-Rad Laboratories)<br>[Microscopy]: Zeiss LSM 880 with Airyscan (Carl Zeiss) or Zeiss Axio Scan Z1 (Carl Zeiss)<br>[HILIC/MS/MS analysis]: UHPLC Nexera liquid chromatography system (Shimadzu Co.) and 5500QTRAP mass spectrometer (AB Sciex Pty. Ltd.)<br>[GS/MS/MS analysis]: Agilent 7890A series gas chromatography system and Agilent 7010B triple-quadrupole mass spectrometer (Agilent Technologies Inc.)<br>[Non-targeted lipidomic analysis]: UltiMate3000 and Q Exactive HF-X Mass Spectrometer (Thermo Fisher Scientific)<br>[Targeted-lipidomic analysis]: ACQUITY Ultra-Performance Convergence Chromatography system and Xevo TQ-XS triple-quadrupole mass spectrometer (Waters)<br>[LC-MS/MS for choline metabolite measurement]: UHPLC Nexera liquid chromatography system (Shimadzu Co.) and 5500QTRAP mass spectrometer (AB Sciex)<br>[Flow cytometer]: BD FACSCelesta (BD Bioscience)<br>[WB]: Fusion Solo7S (Vilber Lourmat)
Data analysis	[qPCR]: CFX Maestro 1.1 (Bio-Rad Laboratories)<br>[Microscopy]: Zen 3.0 (Carl Zeiss)<br>[HILIC/MS/MS analysis]: MultiQuant 3.0 (AB Sciex Pty. Ltd.)<br>[GS/MS/MS analysis]: MassHunter (Agilent Technologies Inc.)<br>[Non-targeted lipidomic analysis]: Expressionist Refiner MS ver. 8.2 (Genedata AG)<br>[Targeted-lipidomic analysis]: TargetLynx (Waters)<br>[LC-MS/MS for choline metabolite measurement]: MultiQuant 3.0 (AB Sciex Pty. Ltd.)

[Flow cytometer]: FlowJo V10 (BD Bioscience)
[WB]: FusionCapt17 (Vilber Lourmat)
[Statistics analysis]: GraphPad Prism 9.3.1 (GraphPad Software)

For manuscripts utilizing custom algorithms or software that are central to the research but not yet described in published literature, software must be made available to editors and reviewers. We strongly encourage code deposition in a community repository (e.g. GitHub). See the Nature Portfolio guidelines for submitting code & software for further information.

Data

Policy information about availability of data

All manuscripts must include a data availability statement. This statement should provide the following information, where applicable:

- Accession codes, unique identifiers, or web links for publicly available datasets
- A description of any restrictions on data availability
- For clinical datasets or third party data, please ensure that the statement adheres to our policy

Source data of each figure used in this study are available in the Supplementary Tables. All other data supporting the findings of this study are available from the corresponding author on reasonable request through the completion of a material transfer agreement with the University of Tokyo and its supplementary materials or in figshare (https://doi.org/10.6084/m9.figshare.23925033).

Research involving human participants, their data, or biological material

Policy information about studies with human participants or human data. See also policy information about sex, gender (identity/presentation), and sexual orientation and race, ethnicity and racism.

Reporting on sex and gender	No sex-based analyses were performed in this study; recruitment was sex-independent.
Reporting on race, ethnicity, or other socially relevant groupings	PFIC1 is an ultrarare pediatric cholestatic liver disease resulting from a genetic defect of ATP8B1. We conducted a nationwide Japanese survey to identify patients with PFIC1. As is true for many rare disease studies, we collected all identified PFIC1 cases and were unable to respecify the sample size. To date, no race- or ethnicity-specific clinical findings have been reported for PFIC1. PFIC1-specific findings can be obtained through analysis of this cohort.
Population characteristics	PFIC1 is an ultrarare pediatric cholestatic liver disease resulting from a genetic defect of ATP8B1. We conducted a nationwide Japanese survey to identify patients with PFIC1 and collected their plasma. Twenty-two PFIC1 patients [pre-liver transplantation (LTx), n = 10; post-LTx, n = 12], 47 patients with other cholestatic diseases (pre-LTx, n = 27; post-LTx, n = 20), and age-matched control individuals (n = 30) were enrolled in this study. The population characteristics are summarized in Table 2.
Recruitment	A nationwide Japanese survey has been conducted to identify patients with PFIC since 2015. The clinical diagnosis of PFIC was based on the presence of unremitting hepatocellular cholestasis with intractable pruritus, jaundice with conjugated hyperbilirubinemia, and elevated serum bile-acid concentrations. Full physical examination, serological, viral, and metabolic markers measurements, imaging, and urine screening were performed to rule out other causes of cholestasis, including hepatitis B and C virus infections, inborn errors in bile-acid synthesis, and ductal origin. These patients were subjected to genetic testing to analyze all exons and flanking intron–exon boundaries of the genes responsible for neonatal/infantile intrahepatic cholestasis, including ATP8B1, by Sanger sequencing, and/or targeted next-generation sequencing. Patients who carried disease-causing mutations in both alleles of ATP8B1 and/or showed ATP8B1 deficiency in phenotypic analysis using peripheral blood monocyte-derived macrophages were diagnosed with PFIC1. Twenty-two PFIC1 patients and 47 patients with a pathogenic variant in genes other than ATP8B1 were identified and enrolled in this study. As is true for many rare disease cohorts, these data may be biased with respect to the socioeconomic status and family screening adherence observed in patients who are eligible for treatment at core hospitals in Japan.
Ethics oversight	The study on human subjects was approved by the institutional review boards at the University of Tokyo, Juntendo University Graduate School of Medicine, Miyagi Children's Hospital, Tsuyama-Chuo Hospital, Yamaguchi University Graduate School of Medicine, Saiseikai Yokohama City Eastern Hospital, Kyoto University Hospital, and National Center for Child Health and Development (permission number: 24-5) and performed in accordance with the 1964 Declaration of Helsinki and its later amendments or comparable ethical standards (as revised in Edinburgh 2000). Informed consent was obtained from all subjects or their parents (when the subjects were under 18) before enrollment in the study.

Note that full information on the approval of the study protocol must also be provided in the manuscript.

Field-specific reporting

Please select the one below that is the best fit for your research. If you are not sure, read the appropriate sections before making your selection.

☒ Life sciences ☐ Behavioural & social sciences ☐ Ecological, evolutionary & environmental sciences

For a reference copy of the document with all sections, see nature.com/documents/nr-reporting-summary-flat.pdf

Life sciences study design

All studies must disclose on these points even when the disclosure is negative.

Sample size	The sample size was chosen based on experimental feasibility, not predetermined by statistical methods. In the mouse experiments, the cohort size was determined by the types of experiments and the availability of animals as littermates. Each experiment was repeated at least three times. For the analysis of human specimens, we used all samples available for us because PFIC1 is an ultrarare pediatric cholestatic liver disease resulting from a genetic defect of ATP8B1. For the cell-based assays, sample sizes were determined in accordance with standard practices in this type of experimental approach (e.g., PMIDs 29104077, 25315773). The detailed sample sizes are listed in the figure legends.
Data exclusions	No data were excluded from the analyses.
Replication	All mouse data are the result of analysis of biological samples obtained from three or more independent replicated experiments. Human samples were studied once due to ethical limitations and the rarity of PFIC1 and related diseases, and each sample was tested in duplicate and produced consistent results. Cell-based assays were performed by at least two independent experiments with cells from different passages. All independent replicates were successful and produced consistent results.
Randomization	In an animal study to evaluate the effects of choline supplementation, mice were randomly allocated to a normal diet or a choline-supplemented diet. In the other animal studies, mice were allocated based on genotype. In the analysis of human specimens, the allocation depended on the type of disease and the presence or absence of surgical treatment (liver transplantation). Randomization was not employed in the cell-based assays since groups were defined before experiments (e.g., EV and ATP8B1).
Blinding	In the mouse studies, the investigators were blinded to genotypic information during the collection of biological samples and their analysis. Blinding was not performed only when intestinal epithelial cells were collected for BBMV preparation, flippase assay, and metabolomic analysis, due to the need for large numbers of mice or financial limitations. In the cell culture experiments, blinding was impossible because transfection with the plasmids by the investigator was required. Blinding is irrelevant in the analysis of human specimens because there was no intervention for the participants.

Reporting for specific materials, systems and methods

We require information from authors about some types of materials, experimental systems and methods used in many studies. Here, indicate whether each material, system or method listed is relevant to your study. If you are not sure if a list item applies to your research, read the appropriate section before selecting a response.

Materials & experimental systems

n/a	Involved in the study	
☐	☒	Antibodies
☐	☒	Eukaryotic cell lines
☒	☐	Palaeontology and archaeology
☐	☒	Animals and other organisms
☒	☐	Clinical data
☒	☐	Dual use research of concern
☒	☐	Plants

Methods

n/a	Involved in the study	
☒	☐	ChIP-seq
☒	☐	Flow cytometry
☒	☐	MRI-based neuroimaging

Antibodies

Antibodies used

Rat anti-F4/80 (Biolegend, 123101, clone: BM8, IHC, 1:100)
Goat anti-GFAP (Abcam, ab53554, polyclonal, IHC, 1:100)
Rabbit anti-αSMA (Cell Signaling Technology, #19245, clone: D4K9N, IHC, 1:100)
Mouse anti-Plin2 (PROGEN, 610102, clone: AP125, IHC, 1:100)
Rabbit anti-MPO (Dako, A0398, polyclonal, IHC, 1:100)
Rabbit anti-Lysozyme (Abcam, ab108508, clone: EPR2994(2), IHC, 1:200)
Rabbit anti-Chga (Abcam, ab15160, polyclonal, IHC, 1:200)
Rabbit anti-Muc2 (Novus Biologicals, NBP1-31231, polyclonal, IHC, 1:200)
Rabbit anti-NHE3 (Novus Biologicals, NBP1-82574, polyclonal, IHC, 1:200)
Goat anti-DPPIV (R&D Systems, AF954, polyclonal, IHC, 1:200)
Rabbit anti-Ezrin (Cell Signaling Technology, 3145, polyclonal, IHC, 1:200)
Rabbit anti-pERM (Cell Signaling Technology, 3726, clone: 48G2, IHC, 1:200)
Mouse anti-E-cadherin (BD Bioscience, 610182, Clone: 36, IHC, 1:200)
Rabbit anti-β-catenin (Cell Signaling Technology, 19807, clone: D2U8Y, 1:200)
Mouse anti-ATP1A1 (Santa Cruz Biotechnology, Sc-21712, clone: C464.6, WB, 1:200)
Rabbit anti-Villin-1 (Cell Signaling Technology, 2369, polyclonal, IHC, 1:100, WB, 1:2000)
Alexa Fluor 488 donkey anti-goat IgG (Thermo Fisher Scientific, A11055, IHC, 1:250)
Alexa Fluor 488 donkey anti-mouse IgG (Thermo Fisher Scientific, A21202, IHC, 1:250)
Alexa Fluor 546 donkey anti-mouse IgG (Thermo Fisher Scientific, A10036, IHC, 1:250)

	Alexa Fluor 488 goat anti-rabbit IgG (Thermo Fisher Scientific, A11008, IHC, 1:250)<br>Alexa Fluor 546 donkey anti-rabbit IgG (Thermo Fisher Scientific, A10040, IHC, 1:250)<br>Alexa Fluor 488 donkey anti-rat IgG (Thermo Fisher Scientific, A21208, IHC, 1:250)
Validation	The specificity of the antibodies was provided by the commercial sources as follows:<br>Rat anti-F4/80 https://www.biolegend.com/ja-jp/products/purified-anti-mouse-f4-80-antibody-4064?GroupID=BLG5319<br>Goat anti-GFAP https://www.abcam.co.jp/products/primary-antibodies/gfap-antibody-ab53554.html<br>Rabbit anti-αSMA https://www.cellsignal.jp/products/primary-antibodies/a-smooth-muscle-actin-d4k9n-xp-rabbit-mab/19245<br>Mouse anti-Plin2 https://www.progen.com//anti-Perilipin-2-N-terminus-mouse-monoclonal-AP125-lyophilized-purified/610102<br>Rabbit anti-MPO https://www.citeab.com/antibodies/3382926-a0398-myeloperoxidase<br>Rabbit anti-Lysozyme https://www.abcam.co.jp/products/primary-antibodies/lysozyme-antibody-epr29942-ab108508.html<br>Rabbit anti-Chga https://www.abcam.co.jp/products/primary-antibodies/chromogranin-a-antibody-ab15160.html<br>Rabbit anti-Muc2 https://www.novusbio.com/products/muc2-antibody_nbp1-31231<br>Rabbit anti-NHE3 https://www.novusbio.com/products/nhe3-slc9a3-antibody_nbp1-82574<br>Goat anti-DPPIV https:/www.rndsystems.com/products/mouse-dppiv-cd26-antibody_af954<br>Rabbit anti-Ezrin https://www.cellsignal.jp/products/primary-antibodies/ezrin-antibody/3145<br>Rabbit anti-pERM https://www.cellsignal.jp/products/primary-antibodies/phospho-ezrin-thr567-radixin-thr564-moesin-thr558-48g2-rabbit-mab/3726<br>Mouse anti-E-cadherin https://www.bdbiosciences.com/ja-jp/products/reagents/microscopy-imaging-reagents/immunofluorescence-reagents/purified-mouse-anti-e-cadherin.610182<br>Rabbit anti-β-catenin https://www.cellsignal.jp/products/primary-antibodies/non-phospho-active-b-catenin-ser45-d2u8y-xp-rabbit-mab/19807ouse anti-ATP1A1 https://www.scbt.com/ja/p/na-k-atpase-alpha1-antibody-c464-6<br>Rabbit anti-Villin-1 https://www.cellsignal.com/products/primary-antibodies/villin-1-r814-antibody/2369<br>Alexa Fluor 488 donkey anti-goat IgG https://www.thermofisher.com/antibody/product/Donkey-anti-Goat-IgG-H-L-Cross-Adsorbed-Secondary-Antibody-Polyclonal/A-11055<br>Alexa Fluor 488 donkey anti-mouse IgG https://www.thermofisher.com/antibody/product/Donkey-anti-Mouse-IgG-H-L-Highly-Cross-Adsorbed-Secondary-Antibody-Polyclonal/A-21202<br>Alexa Fluor 546 donkey anti-mouse IgG https://www.thermofisher.com/antibody/product/Donkey-anti-Mouse-IgG-H-L-Highly-Cross-Adsorbed-Secondary-Antibody-Polyclonal/A10036<br>Alexa Fluor 488 goat anti-rabbit IgG https://www.thermofisher.com/antibody/product/Goat-anti-Rabbit-IgG-H-L-Cross-Adsorbed-Secondary-Antibody-Polyclonal/A-11008<br>Alexa Fluor 546 donkey anti-rabbit IgG https://www.thermofisher.com/antibody/product/Donkey-anti-Rabbit-IgG-H-L-Highly-Cross-Adsorbed-Secondary-Antibody-Polyclonal/A10040<br>Alexa Fluor 488 donkey anti-rat IgG https://www.thermofisher.com/antibody/product/Donkey-anti-Rat-IgG-H-L-Highly-Cross-Adsorbed-Secondary-Antibody-Polyclonal/A-21208

Eukaryotic cell lines

Policy information about cell lines and Sex and Gender in Research

Cell line source(s)	HEK293T (CRL-11268) cells and CHO-K1 (CCL-61) cells were purchased from the American Type Culture Collection (Manassas, VA).
Authentication	All cell lines used in this study were authenticated by STR profiling in ATCC.
Mycoplasma contamination	All cell lines were tested negative for mycoplasma contamination.
Commonly misidentified lines<br>(See ICLAC register)	No commonly misidentified cell lines were used.

Animals and other research organisms

Policy information about studies involving animals; ARRIVE guidelines recommended for reporting animal research, and Sex and Gender in Research

Laboratory animals	Atp8b1flox/flox mice were generated as follows:<br>C57BL/6J female mice were mated with C57BL/6J male mice and mixtures of pX330-mC and pflox-Atp8b1 were microinjected into the zygotes collected from oviducts. The surviving zygotes were then implanted into the oviducts of pseudopregnant ICR females, and newborns were obtained. ICR and C57BL/6J mice were purchased from Charles River Laboratories International, Inc. (Yokohama, Japan).<br><br>Atp8b1flox/flox mice were crossed with villin-Cre transgenic mice (Stock No. 021504; The Jackson Laboratory, Bar Harbor, ME) and villin-Cre/ERT2 transgenic mice (Stock No: 020282; The Jackson Laboratory) to obtain Atp8b1IEC-KO mice and Atp8b1Tax-iIEC-KO mice, respectively. Newborn and four-week-old Atp8b1IEC-KO mice and littermate Atp8b1flox/flox mice were analyzed. Eight-week-old Atp8b1Tax-iIEC-KO mice and littermate Atp8b1flox/flox mice were treated daily for 4 days with 1 mg Tax intraperitoneally and then analyzed.
Wild animals	No wild animals were used in this study.
Reporting on sex	In this study, both male and female patients were recruited and analyzed for the human study, while males were used in the mouse

	study. This is in line with previous studies on steatosis in mice, which have primarily used males because of the metabolic effects of female hormones (PMID: 36646715, PMID: 36934083).
Field-collected samples	This study did not use samples collected from the field.
Ethics oversight	All mouse experiments were approved by and performed in accordance with the guidelines of the animal experiment committee of the University of Tokyo and the University of Tsukuba (permission number: P29-24).

Note that full information on the approval of the study protocol must also be provided in the manuscript.
